# Design, Dimerization, and Recombinant Production of MCh-AMP1–Derived Peptide in *Escherichia coli* and Evaluation of Its Antifungal Activity and Cytotoxicity

**DOI:** 10.3389/ffunb.2021.638595

**Published:** 2021-04-26

**Authors:** Sima Sadat Seyedjavadi, Soghra Khani, Jafar Amani, Raheleh Halabian, Mehdi Goudarzi, Hamideh Mahmoodzadeh Hosseini, Ali Eslamifar, Masoomeh Shams-Ghahfarokhi, Abbas Ali Imani Fooladi, Mehdi Razzaghi-Abyaneh

**Affiliations:** ^1^Department of Mycology, Pasteur Institute of Iran, Tehran, Iran; ^2^Applied Microbiology Research Center, Systems Biology and Poisonings Institute, Baqiyatallah University of Medical Sciences, Tehran, Iran; ^3^Department of Microbiology, School of Medicine, Shahid Beheshti University of Medical Sciences, Tehran, Iran; ^4^Department of Clinical Research, Pasteur Institute of Iran, Tehran, Iran; ^5^Department of Mycology, Faculty of Medical Sciences, Tarbiat Modares University, Tehran, Iran

**Keywords:** antifungal activity, cytotoxicity, antimicrobial peptide, *Aspergillus*, *Candida*, dimeric antifungal peptides, bacterial expression system, *Matricaria chamomilla* (chamomile)

## Abstract

Fungal species resistant to current antifungal agents are considered as a serious threat to human health, the dilemma that has dragged attentions toward other sources of antifungals such as antimicrobial peptides (AMPs). In order to improve biological activity of a recently described antifungal peptide MCh-AMP1 from *Matricaria chamomilla* flowers, MCh-AMP1dimer (DiMCh-AMP1), containing 61 amino acid residues connected by flexible linker (GPDGSGPDESGPDES), was designed and expressed in *Escherichia coli*, and its structure was analyzed using bioinformatics tools. DiMCh-AMP1 synthetic gene was cloned into pET-28a expression vector, which was then used to transform *E. coli* BL21 (DE3) strain. His-tag purification was achieved using metal-chelate affinity chromatography. Because there is no methionine residue in the DiMCh-AMP1 sequence, cyanogen bromide was successfully used to separate the target product from the tag. Reverse-phase high-performance liquid chromatography was used as the final step of purification. Results showed that recombinant peptide was produced in considerable amounts (0.9 mg/L) with improved antifungal activity toward both yeasts and molds compared to its monomeric counterpart. The minimum inhibition concentration and minimum fungicidal concentration values of DiMCh-AMP1 against *Candida* and *Aspergillus* species were reported in the range of 1.67–6.66 μM and 3.33–26.64 μM, respectively. Our results showed that while antifungal activity of dimerized peptide was improved considerably, its cytotoxicity was decreased, implying that DiMCh-AMP1 could be a potential candidate to design an effective antifungal agent against pathogenic yeasts and molds.

## Introduction

With increasing of debilitating diseases that affect human immune system in recent years, more people are now at risk of life-threatening fungal infections, making it a growing human health concern (Rodrigues and Nosanchuk, 2020). Aside from a few number of antifungal agents to combat such infections, uncontrolled administration of such drugs caused species resistant to antifungal agents and, as a subsequence, narrowed the control of fungal infections (Beardsley et al., 2018). To overcome this dilemma, antimicrobial peptides (AMPs) and their analogs are in the center of attention for therapeutic purposes (Cruz et al., 2014). AMPs are not only small in size (<100 amino acid residues), but also they are cationic, amphipathic, variable in length, and sequence with diverse structures originated from animals, plants, bacteria, and fungi (Pushpanathan et al., 2013; Malmsten, 2014; Silva et al., 2014). It has been shown that AMPs, as the main parts of the innate immunity system, play a significant role in fighting pathogens (Seo et al., 2012).

The clinical application of AMPs has already faced different problems, which should be overcome before introducing them as new agents for therapeutic purposes. Hence, different approaches were used to promote the stability, efficacy, and selectivity of AMPs (Wang P. et al., 2010; Crusca Jr et al., 2011; Luong et al., 2020). According to the current knowledge, the original AMPs have potential dimerization capability, which can improve their antimicrobial potency, selectivity, and solubility (Falciani et al., 2007; Güell et al., 2012; Hernandez-Gordillo et al., 2014). Therefore, dimeric AMPs can be considered as novel antimicrobial agents with improved activity and selectivity.

To dimerize peptides, linkers are used to attach two molecules that can conserve or improve their parental biological activity. Several studies have demonstrated that linker flexibility plays key role in biological activity of dimerized AMPs (Lorenzón et al., 2012; Reddy Chichili et al., 2013; Jamasbi et al., 2014). As chemical synthesis is highly expensive, industrial manufacturers are more interested in recombinant expression of peptides that benefit from preliminary equipment and the ease in scaling-up of the methods. *Escherichia coli* is the most popular expression system, as it is time-efficient, its DNA is easy to manipulate, and expression level is high (Li, 2011).

In our previous study, MCh-AMP1 (LSVKAFTGIQLRGVCGIEVKARG) (2,402.369 Da), an antifungal peptide, was isolated from *Matricaria chamomilla* and found to be active against *Candida* and *Aspergillus* species (Seyedjavadi et al., 2019). Additionally, MCh-AMP1 had a negligible hemolytic activity for human erythrocytes. In the present study, the dimerization of MCh-AMP1 (DiMCh-AMP1) was designed with a flexible linker (GPDGSGPDESGPDES), aiming to increase antifungal activity and to decrease the cytotoxicity against animal cells. The DiMCh-AMP1 gene was synthesized and expressed in *E. coli* BL21 (DE3), and its antifungal and hemolytic activities were determined.

## Materials and Methods

All the experiments of the present study involving human samples were carried out under an ethical statement (IR NIMAD REC 1396 121) from the National Institute for Medical Research Development, IRAN.

### Bacterial and Fungal Strains and Vector

For peptide expression, *E. coli* BL21 (DE3) strain was used. To express peptide construct, the expression vector pET-28a (+) was utilized. *Candida albicans* ATCC 10231, *Candida krusei* DSM 70079, *Candida glabrata* ATCC 90030, *Aspergillus fumigatus* Af 293, *Aspergillus niger* ATCC 9029, and *Aspergillus flavus* PFCC 100 were obtained from the Pathogenic Fungi Culture Collection of the Pasteur Institute of Iran (http://fa1.pasteur.ac.ir/MBankResult.aspx).

### Design and Secondary and Tertiary Structure Analyses of Chimeric Construct

To obtain DiMCh-AMP1 (N-terminal to C-terminal connection), the amino acid sequences were fused together using the proper linker. In order to select the best linker, the secondary and tertiary structures of the various linkers with maximum efficiency were analyzed by GOR4 (http://npsa-pbil.ibcp.fr/cgi-bin/npsa_automat.pl?page=npsa_gor4.html) (Garnier, 1998) and I-TASSER (http://zhanglab.ccmb.med.umich.edu/I-TASSER/) online server tools (Zhang, 2008). The linker causing less conformational changes in the native MCh-AMP1 structure, as assessed *in silico*, was then selected. The DiMCh-AMP1 construct was back-translated and optimized based on bacterial expression host, *E. coli* codon usage by Gene Designer-v1 software. After codon optimization, Met codon site for cyanogen bromide (CNBr) cleavage and *Bam*HI restriction site were added in 5′ end of the chimeric gene, and two stop codons (TAA) and *Hin*dIII were added in the 3′ end of the chimeric gene. DNA sequence of DiMCh-AMP1 is as follows:

5′-GGATCCATGCTGTCTGTTAAGGCTTTTACTGGTATTCAACTGCGTGGTGTTTGCGGTATTGAAGTAAAAGCTCGTGGCGGTCCGGATGGTTCTGGTCCGGATGAAAGCGGCCCAGACGAATCTCTGTCTGTTAAAGCATTTACCGGTATCCAGCTGCGTGGTGTATGCGGTATCGAAGTCAAGGCGCGCGGCTAAAAGCTT-3′.

### Chimeric Construct Characterization

The physicochemical properties of MCh-AMP1 and DiMCh-AMP1 were analyzed using ExPASy ProtParam tool (http://expasy.org/tools/protparam.html).

### Prediction of RNA Secondary Structure

The secondary mRNA structure and mRNA stability of the chimeric genes were predicted by mfold web server (http://mfold.rna.albany.edu/?q=mfold/RNA Folding-Form) (Zuker, 2003).

### Construction of pET-28a (+)-DiMCh-AMP1 Plasmid

The DNA sequence of DiMCh-AMP1 was chemically synthesized (Biomatik, Canada) and then cloned into the pET-28a (+) vector (Merck, Germany) using *Bam*HI and *Hin*dIII restriction sites. The plasmid contained a pro-peptide, a 6His-tag, a Met codon for CNBr cleavage, and a DiMCh-AMP1gene. The construction was named pET-28a (+)-DiMCH-AMP1 conferring kanamycin resistance. DNA sequencing analysis confirmed the recombinant plasmid pET-28a (+)-DiMCH-AMP1. The heat shock transformation method was used to transform the recombinant plasmid (pET-28a (+)-DiMCH-AMP1) into competent *E. coli* BL21 (DE3). The colony polymerase chain reaction (PCR) was employed to confirm the cells that positively transformed. To perform PCR, T7 universal primers were used, and the PCR products were run on 1.5% agarose gel by electrophoresis.

### Expression of the Recombinant Peptide

A fresh colony of transformed *E. coli* BL21 (DE3) with expression vector was inoculated into a 50 mL Luria–Bertani (LB) medium containing 50 mg/L kanamycin and incubated on a shaker incubator at 37°C for 12 h. Then, 10 mL of the culture was transferred to 500 mL of a fresh LB medium containing 50 mg/L kanamycin and cultured at 37°C on a 200-rpm shaker until the optical density (OD 600) reached 0.6 to 0.8. The peptide expression was induced by IPTG (isopropyl-β-d-thiogalactoside) (Sigma, St. Louis, MO, USA) to a final concentration of 1 mM, and the culture was incubated at 37°C in a shaker for 4 h. The culture was centrifuged at 5,000 rpm at 4°C for 10 min to harvest the cells. The bacterial pellets were resuspended in the binding buffer (20 mM NaH_2_PO_4_, 500 mM NaCl, 20 mM imidazole, pH 7.5) and were lysed by mechanical glass bead disruption. The lysate was centrifuged at 14,000 rpm for 20 min at 4°C to collect the supernatant. The 15% sodium dodecyl sulfate–polyacrylamide gel electrophoresis (SDS-PAGE) was employed to detect extracted proteins; then, Coomassie brilliant blue R-250 was used to stain the proteins. To optimize the highest protein expression, different conditions of OD_600_ values in presence of various IPTG concentrations (0.4, 0.6, 0.8, and 1.0 mM), incubation times (1, 2, and 4 h), and temperatures (30°C and 37°C) were examined.

### Purification of the Recombinant Peptide

To purify the recombinant DiMCh-AMP1, a nickel-nitrilotriacetic acid resin–packed column was used under native conditions. The column was equilibrated by binding buffer, and the extracted proteins from recombinant expression in *E. coli* were loaded on the column and eluted by a gradient of the imidazole concentration (0–100%) by an elution buffer (20 mM NaH_2_PO_4_, 500 mM NaCl, 500 mM imidazole; pH 7.5). Fractions were analyzed on 15% SDS-PAGE, and the fractions containing DiMCh-AMP1 were pooled and dialyzed overnight at 4°C against deionized water. Protein concentration was determined by bovine serum albumin (Sigma) as standard (Bradford, 1976).

### Confirmation and Characterization of the Recombinant Peptide by Western Blot

To confirm expression in *E. coli*, the purified DiMCh-AMP1 containing fusion protein was transferred to a polyvinylidene fluoride membrane using transfer buffer (39 mM glycine, 48 mM Tris base, 0.037% SDS, and 20% methanol) by the Bio-Rad Mini Protean II System (Philadelphia, PA, USA). The blocking buffer of 3% skim milk/Tris-buffered saline (137 mM NaCl, 2.7 mM KCl, and 4.3 mM Na_2_HPO_4_, pH 7.3) was added to the membrane at room temperature for 2 h. Horseradish peroxidase (HRP)–conjugated mice anti–His-tag antibody (Sigma) prepared in the TBS/T (Tween-buffered saline containing 0.05% Tween 20) was then added to the membrane by a 1:1,000 dilution ratio following gentle shaking at room temperature for 2 h. The membrane was washed in triplicate afterward with TBS/T, and then an HRP staining solution (diaminobenzidine) was used to visualize the proteins.

### Cleavage of DiMCh-AMP1

To separate fusion protein from the peptide, purified DiMCh-AMP1 containing fusion protein was centrifuged for 30 min at 4°C after dialysis. After discarding the supernatant, the pellet was incubated with a CNBr solution (HCl >0.5 M and CNBr with a molar ratio to protein as 100:1) in dark at room temperature for 18 h and dried under the vacuum by lyophilization. To analyze the cleavage, 16.5% tricine-Tris SDS-PAGE was used (Gels et al., 1987).

### Purification of DiMCh-AMP1by High-Performance Liquid Chromatography

To purify the peptide after cleavage, C18 Reverse Phase-High Performance Liquid Chromatography (RP-HPLC) on the Knauer HPLC system (Knauer, Germany) was used by a TSK gel ODS-120Ts column (C18 column, 7.8 × 300 mm; Tosoh, Tokyo, Japan). The column was equilibrated with 10% (vol/vol) acetonitrile containing 0.1% (vol/vol) trifluoroacetic acid (TFA) and eluted with a gradient step from 10 to 40% (vol/vol) acetonitrile for 1 h at a flow rate of 1 mL/min. Fractions detected at 220-nm wavelength were collected and lyophilized. A 16.5% tricine-Tris SDS-PAGE was used to analyze the peaks. The fractions containing pure DiMCh-AMP1 were collected and lyophilized. The protein content of the purified peptide was determined (Bradford, 1976).

### Assay of Antifungal Activity

The minimum inhibition concentration (MIC) of the recombinant peptide was determined by microdilution method in 96-well microplates according to Li et al. (2015) with some modifications. For this purpose, the recombinant peptide was provided in triplicate sets of 2-fold serial dilutions (0.83–53.23 μM) up to a final volume of 100 μL in a 96-well microplate. Then, 100 μL of fungal suspension (10^5^ cells/mL) was added to each well, and the plate was incubated at 35°C for 24–48 h. The pure broth alone and the broth with the inoculum suspension were used as the negative and positive controls, respectively. The lowest concentration that inhibited fungal growth was referred to as the MIC, which was determined by visual inspection. To determine the minimum fungicidal concentration (MFC), a 20-μL volume of the content of each well with no visible fungal growth was cultured on Sabouraud dextrose agar plates. The plates were incubated at 35°C for 24–72 h, and the lowest concentration that killed at least 99.9% of the primary inoculums was considered as MFC.

### Hemolysis Assay

Hemolytic activity of the peptide was determined according to Asoodeh et al. (2012) by using human red blood cells obtained from Blood Transfusion Center, Tehran, Iran. To provide a 4% (vol/vol) solution, phosphate-buffered saline (PBS) (pH 7.4) was used, and then the solution was exposed to different concentrations of the recombinant peptide (0.83–105.56 μM) at 37°C for 1 h. Based on the absorption rate, hemoglobin release was determined using an enzyme-linked immunosorbent assay reader at 567 nm by the following formula:


$$\begin{array}{l} \text{Hemolysis}(\%)=[\left(\text{OD of test-OD of negative control}\right)/\\ \;\left(\text{OD of positive control-OD of negative control}\right)]\\ \;\times 100. \end{array}$$

The control samples were PBS (negative control) and 1% Triton X-100 (positive control). All the experiments were carried out in triplicate.

### Statistical Analyses

Data were analyzed by GraphPad Prism 6.0, and the results were expressed as mean ± standard deviation.

## Results

### Choice of the Best Order of Linker Determinants in the Chimeric Peptide

The secondary and tertiary predicted structures of DiMCh-AMP1 were analyzed to determine the best linker among available flexible linkers. Different sequences were submitted to secondary structure prediction servers and also to I-TASSER for three-dimensional (3D) structure predictions (data not shown in details). Likewise, the peptide with native structure was superimposed onto its coordinates in the chimeric peptide, and its Root-Mean-Square Deviation (RMSD) was calculated. Finally, GPDGSGPDESGPDES sequence was selected as the linker to retain the flexibility of the construct. RMSD of the native peptide in the chimeric peptide was 1.76 Å.

### Secondary and Tertiary Structure Prediction

**Figure 1:**
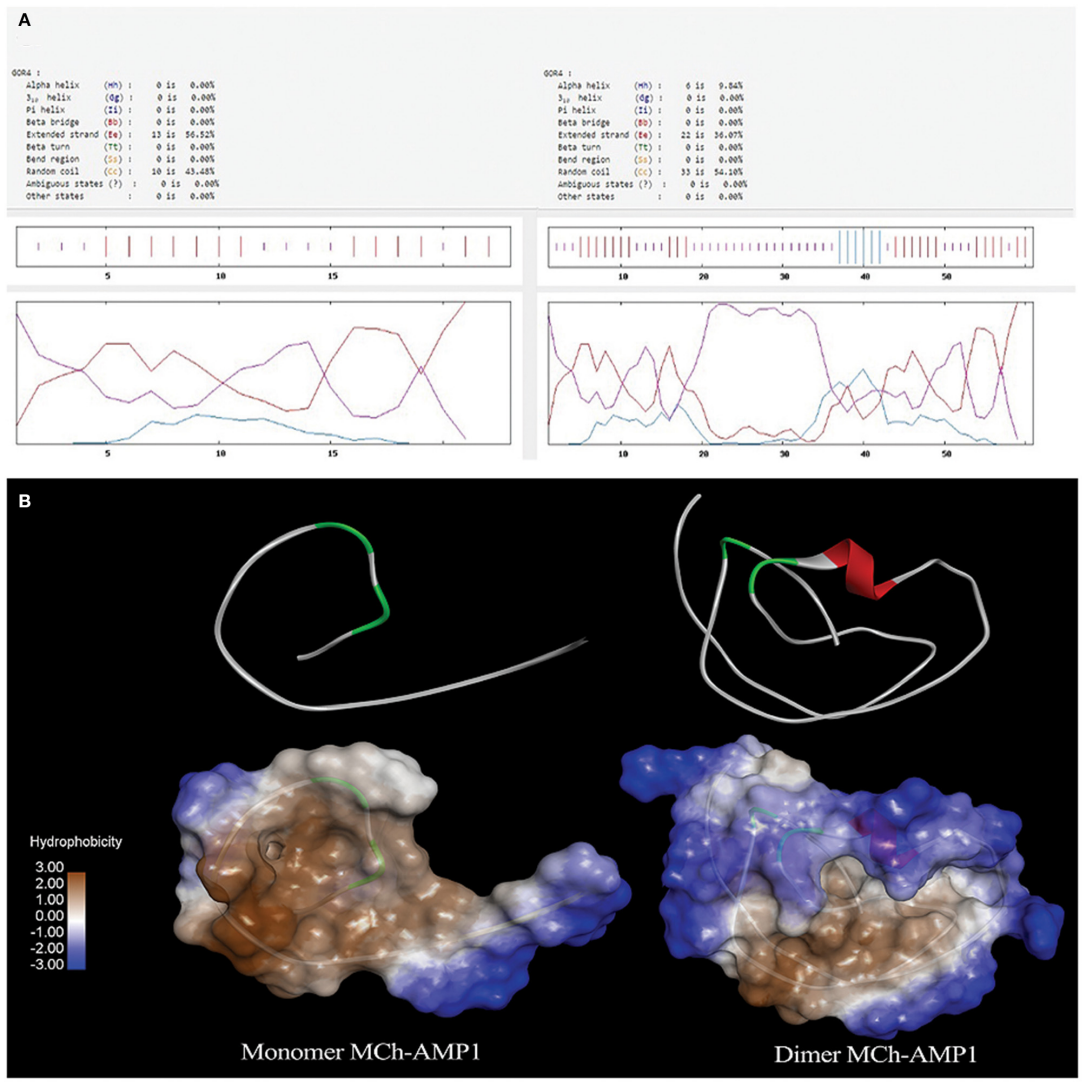
**(A)** Result of secondary structure prediction for MCh-AMP1 and DiMCh-AMP1 peptides by GOR4 software. **(B)** I-TASSER server was used to predict the 3D structure of the MCh-AMP1 and DiMCh-AMP1 peptides. Results were read by Accelrys Discovery Studio Visualizer 2.0 software.

GOR4 was used to predict the secondary structure of DiMCh-AMP1. The comparison of the MCh-AMP1 secondary structure and its coordinate in the DiMCh-AMP1 showed the best prediction capability for the GOR4 server of MCh-AMP1 and DiMCh-AMP1 secondary structures as 9.8% helix, 36% extended strand, and 54% random coil in DiMCh-AMP1peptide structure. A comparison of DiMCh-AMP1 secondary structure with MCh-AMP1 peptide showed 56% extended strand and 43.4% random coil (Figure 1A). I-TASSER server as an *in silico* method was used to predict the 3D structure of MCh-AMP1 and DiMCh-AMP1 (Figure 1B). The 3D models were validated by available bioinformatics tools. The C-scores of model 1 of MCh-AMP1 and DiMCh-AMP1 were −1.66 and −2.87, and those of RMSD model 1 of MCh-AMP1 and DiMCh-AMP1 were 4.3 and 8.5, respectively. According to the findings, the model had a high confidence and correct topology.

### Primary Sequence Analysis

The designed DiMCh-AMP1 contained 61 amino acid residues. The physicochemical characteristics of DiMCh-AMP1 and MCh-AMP1 peptides are shown in Table 1. According to the ProtParam and ADP2 results, the DiMCh-AMP1 molecular weight was 6,172 Da with pI of 8.02.

**Table 1:** Physicochemical properties of MCh-AMP1 and DiMCh-AMP1 are shown.

**Peptides**	**Sequence**	**Molecular**	**Net charge**	**Boman**	**Total hydrophobic**	**Theoretical**
		**weight**	**(pH 7)**	**index**	**ratio (%)**	**pI**
MCh-AMP1	LSVKAFTGIQLRGVCGIEVKARG	2,405	+3	0.68	47.0	10.05
DiMCh-AMP1	LSVKAFTGIQLRGVCGIEVKARGGPDGSGPDESGPDE SLSVKAFTGIQLRGVCGIEVKARG	6,172	+1	1.27	36.0	8.02

### RNA Secondary Structure

**Figure 2:**
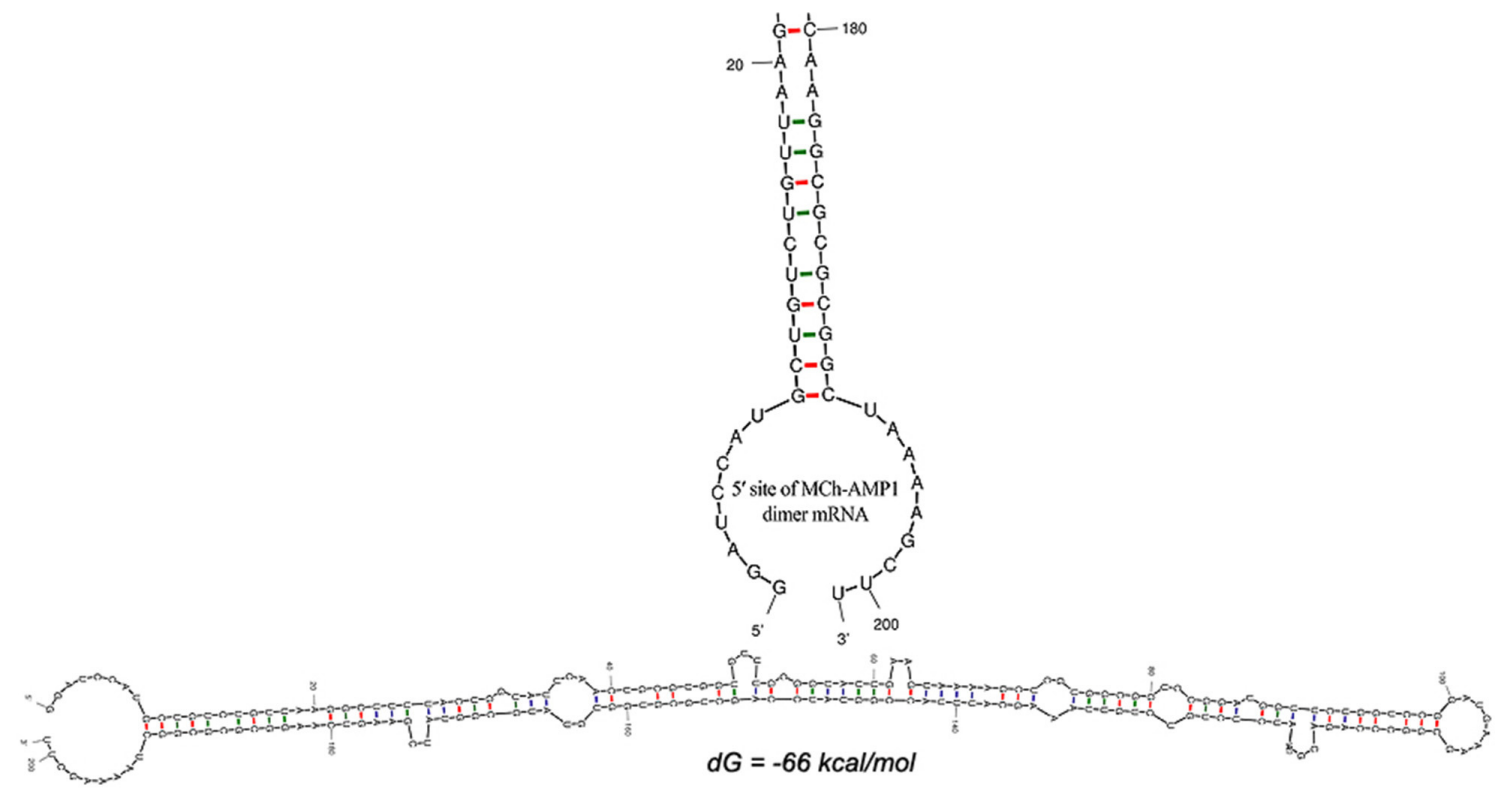
Secondary structure prediction by mfold server shows the optimal mRNA secondary structure of DiMCh-AMP1. Predicted structure has no long stable hairpin and pseudo knot at 5′ site of mRNA. Δ*G* = −66 kcal/mol, mfold tool.

The DiMCh-AMP1 peptide was reverse-translated *in silico* using codons optimized for *E. coli* with the Gene Designer program. After the optimization of the DiMCh-AMP1, coding sequences based on the preferential codon usage of *E. coli*, mfold, and RNA-fold web servers (http://www.unafold.org) were used to predict secondary structure of the DiMCh-AMP1 mRNA. Results are shown in Figure 2. The free energy of thermodynamic ensemble related to this structure is −66 kcal/M.

### Construction of the Recombinant Plasmid

**Figure 3:**
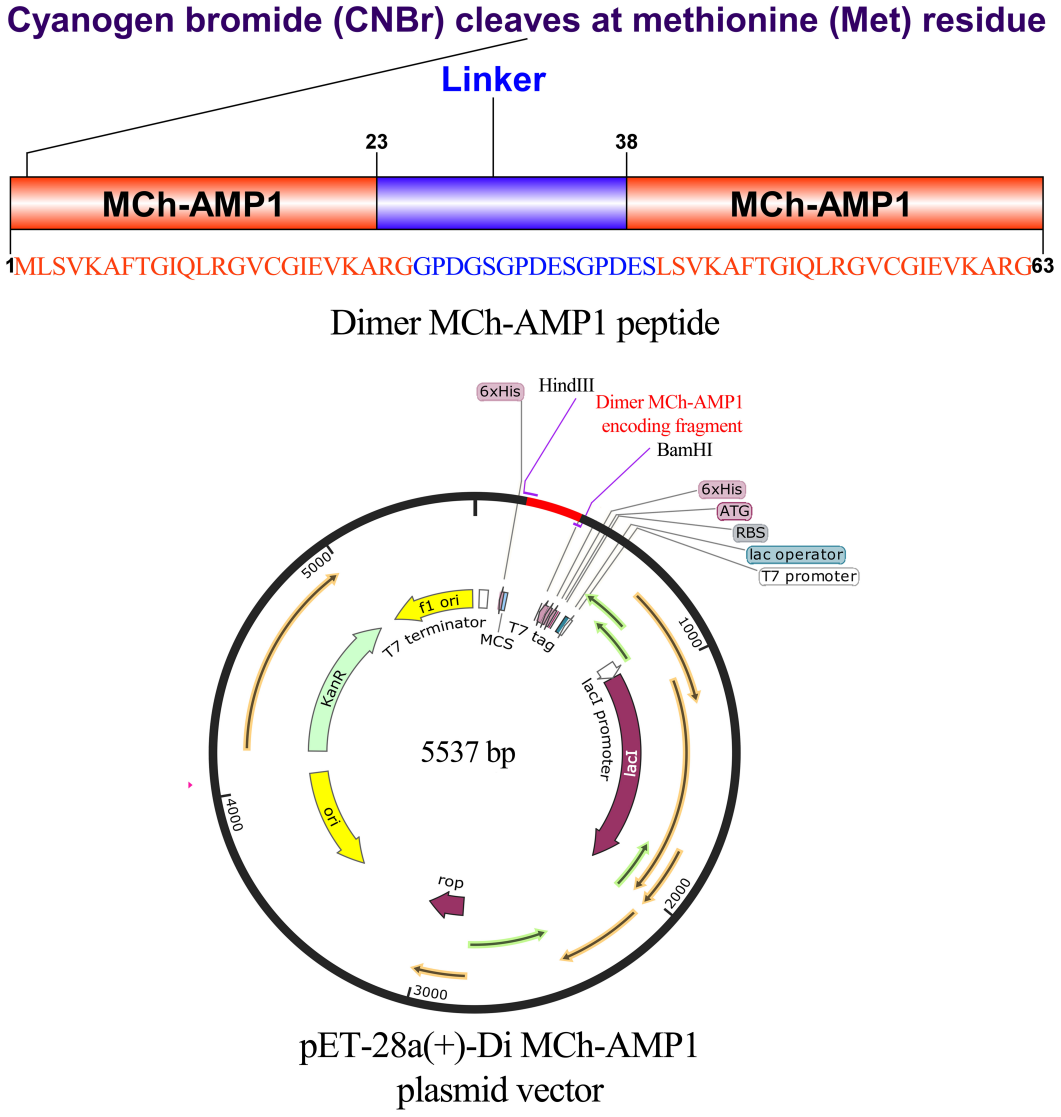
Gene construction of the plasmid carrying pET28a(+)-DiMCh-AMP1. The organization of the gene and amino acid sequences in pET28a(+)-DiMCh-AMP1. Schematic representation of the expression vector.

The expression vector construction process is depicted in Figure 3. As soon as the DiMCh-AMP1 gene was synthetized, to facilitate the recombinant proteins expression, the gene encoding DiMCh-AMP1 was subcloned into the expression vector pET-28a(+) harboring pro-peptide structure. The expression vector pET-28a(+) coded 96 residues including a pro-peptide [four for initial protein translation (MRGS), six for the His-tag, 24 extra residues, and one for the methionine for CNBr cleavage region] and the 61 residues of the mature DiMCh-AMP1 peptide–pET-28a(+)-DiMCh-AMP1. In brief, the gene cloned into the plasmid pET28a(+) produced a ~9.8-kDa pro-peptide (DiMCh-AMP1).

### Expression and Affinity Purification in *E. coli* BL21DE3

**Figure 4:**
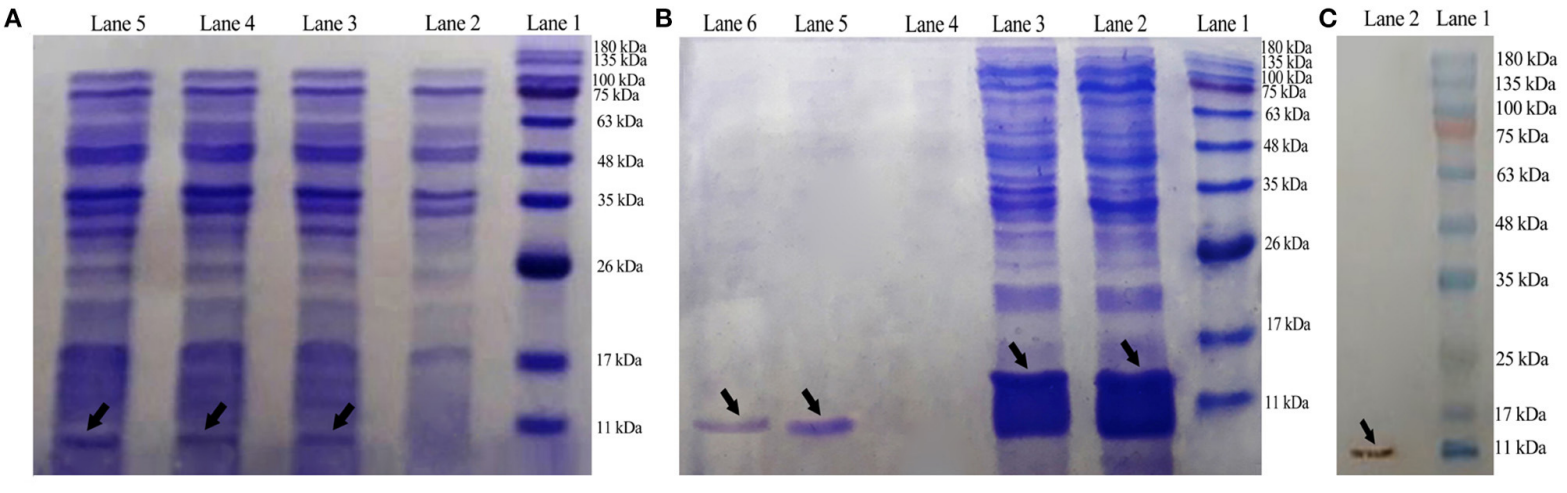
Expression, Western blot analysis, purification, and characterization of recombinant MCh-AMP1. **(A)** SDS-PAGE showing the expression of pET28a(+)-DiMCh-AMP1: Lane 1, molecular weight marker (MWM); Lane 2, cells without IPTG induction; Lane 3, cells with IPTG induction after 1 h; Lane 4, cells with IPTG induction after 2 h; Lane 5, cells with IPTG induction after 4 h. **(B)** Purification of the recombinant DiMCh-AMP1 peptide with 6X-His-tagged from pET28a: Lane 1, protein weight marker; Lane 2, flow through; Lane 3, washing column with wash buffer; Lanes 5 and 6, purified protein after elution with elution buffer. **(C)** Western blot analysis of DiMCh-AMP1 using anti−6X-His-tag antibodies: Lane 1, molecular weight marker; Lane 2, DiMCh-AMP1.

By using the T7 promoter under the IPTG induction, *E. coli* BL21 (DE3) was transformed with plasmid pET-28a(+)-DiMCh-AMP1 for the expression. SDS-PAGE was used to analyze the expression of the constructed vector including the 9.8-kDa recombinant peptide in the presence or absence of IPTG (Figure 4A). In BL21 cells, Lanes 3 to 5 indicate the presence of an intense band (shown by an arrow) with a ~9.8-kDa molecular weight as matched by the DiMCh-AMP1 (~9.8 kDa), which is more intense in Lane 5 (after 4 h).

Most of the expressed DiMCh-AMP1 was soluble in the cell lysate supernatant (data not shown). DiMCh-AMP1 was purified by the Ni-NTA column, in a 250-mM imidazole elution buffer. Figure 4B shows the SDS-PAGE results of the purification of DiMCh-AMP1 and Lanes 5 and 6 show an intense band of the purified ~9.8 kDa DiMCH-AMP1. The DiMCh-AMP1 expression in BL21 (DE3) was 2.1 mg/L after purification. The expression level reached its highest level after 4 h of 1 mM IPTG induction (data not shown).

### Western Blotting Analysis

Western blot analysis was performed to detect DiMCh-AMP1 through the His-tag using anti–His-tag antibodies HRP-conjugated. The result in Figure 4C indicates a ~9.8-kDa band that corresponds to DiMCh-AMP1 expression as observed by 15% SDS-PAGE analysis.

### Cleavage and Purification of DiMCh-AMP1

**Figure 5:**
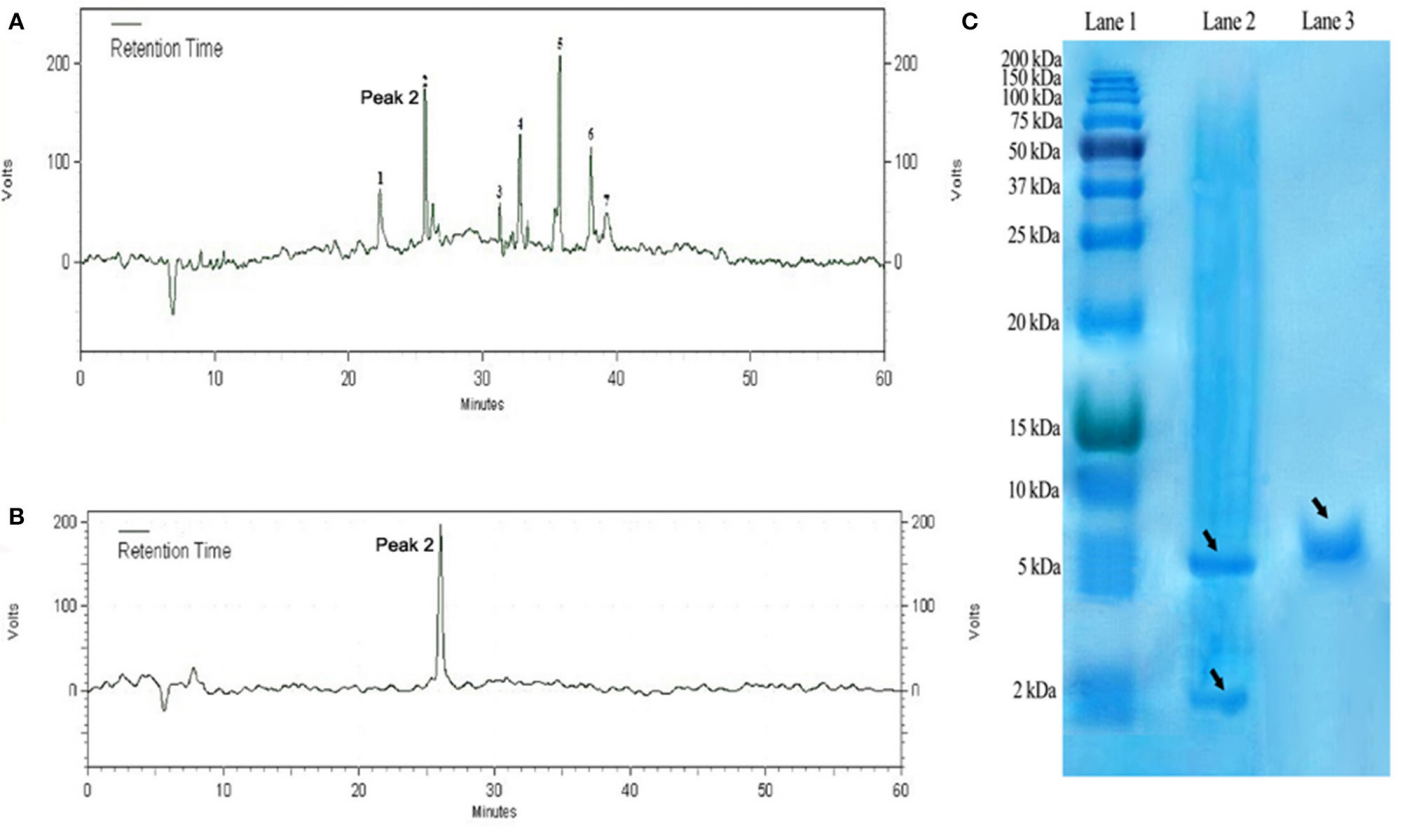
Purification steps of DiMCh-AMP1, which was eluted at 26 min. **(A)** Reversed-phase HPLC purification of DiMCh-AMP1 after cleavage by CNBr. **(B)** The purity of the labeled peak 2. **(C)** SDS-PAGE analysis of the HPLC fractions: Lane 1, protein marker; Lane 2, mixture after CNBr cleavage; Lane 3, peak DiMCh-AMP1 collected from HPLC. Proteins were stained with Coomassie brilliant blue. The MW of the purified DiMCh-AMP1 was found to be ~6,172 Da based on the SDS-PAGE analysis. The theoretical MW of DiMCh-AMP1 analysis was calculated as 6,172.04 Da.

The purified DiMCh-AMP1 by affinity chromatography was digested at methionine residues with CNBr. SDS-PAGE was used to verify the digestion by CNBr. The protein mixture digested by CNBr was purified with RP-HPLC based on a water/acetonitrile gradient containing 0.1% TFA. HPLC chromatograms are given in Figures 5A,B. The retention time of DiMCh-AMP1 was 26 min verified by SDS-PAGE (Figure 5C, Lane 3). The eluted DiMCh-AMP1 weighed ~6,172 Da based on the 16.5% tricine-Tris gel. The yield was 0.9 mg of DiMCh-AMP1 per liter of *E. coli* cell culture in LB medium.

### Assay of Antifungal Activity

The antifungal activity of recombinant DiMCh-AMP1 determined via MIC assay is shown in Table 2. The recombinant DiMCh-AMP1 was active against the yeasts and filamentous fungi. As shown in Table 2, the MICs of recombinant DiMCh-AMP1 ranged from 1.67 to 3.33 and 3.33 to 6.66 μM for *Candida* and *Aspergillus* species, respectively. The recombinant DiMCh-AMP1 had fungicidal activity with MFC values ranging from 3.33 to 26.64 μM. Compared to DiMCh-AMP1, synthetic MCh-AMP1 had MIC and MFC values ranging from 3.33 to 13.32 and 13.32 to 26.64 μM against *Candida* and *Aspergillus* species, respectively.

**Table 2:** The MIC and MFC values of MCh-AMP1 and DiMCh-AMP1 for tested fungal strains.

**Fungal strain**	**MIC (μM)**		**MFC (μM)**	
	**MCh-AMP1**	**DiMCh-AMP1**	**MCh-AMP1**	**DiMCh-AMP1**
*Candida albicans* ATCC 10231	6.66	3.33	13.32	6.66
*Candida glabrata* ATCC 90030	3.33	1.67	26.64	3.33
*Candida krusei* DSM 70079	6.66	3.33	13.32	3.33
*Aspergillus fumigatus* Af293	13.32	3.33	26.64	13.32
*Aspergillus flavus* PFCC 100	6.66	6.66	13.32	26.64
*Aspergillus niger* ATCC 9029	6.66	3.33	26.64	6.66

### Hemolytic Activity

**Figure 6:**
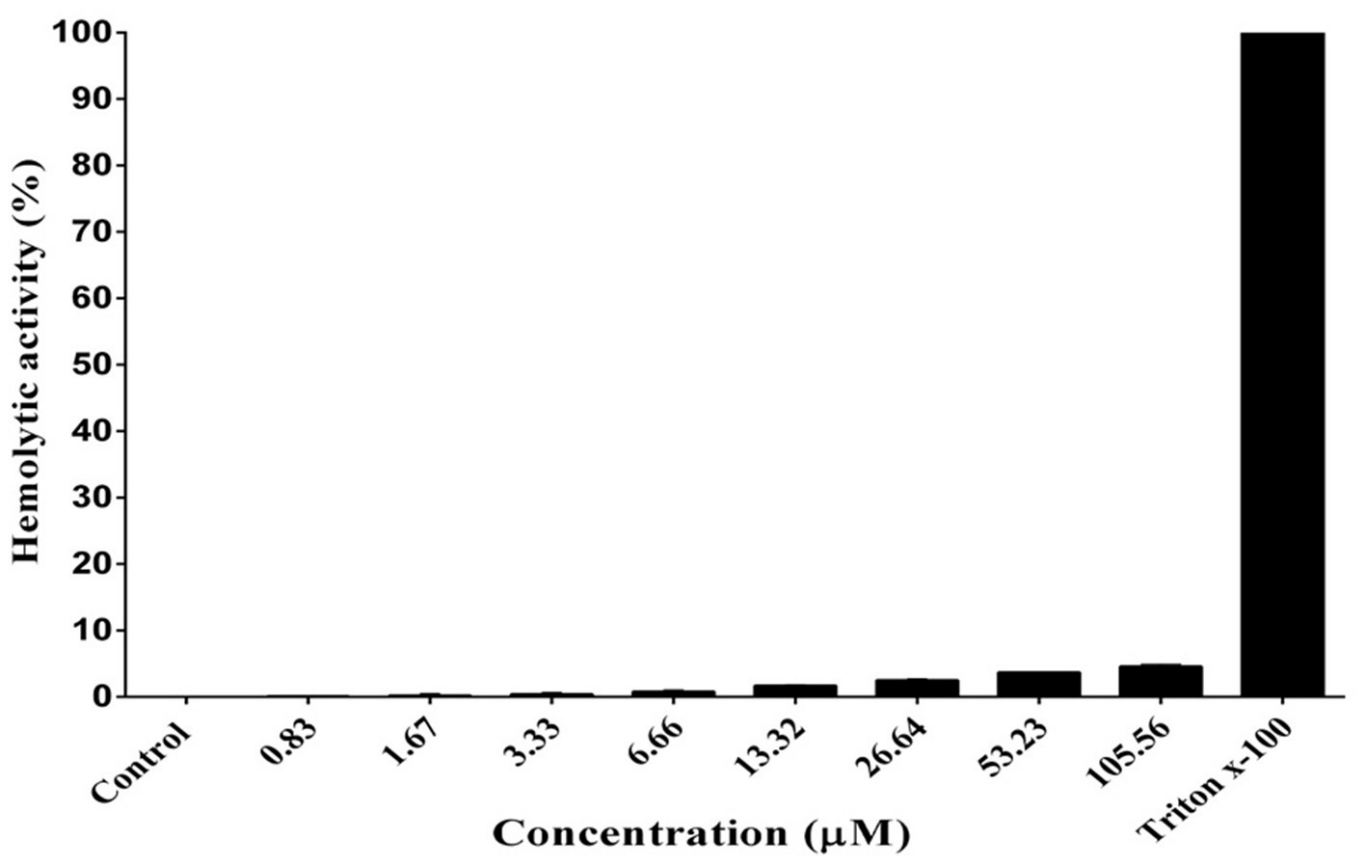
Hemolytic activity of DiMCh-AMP1 against human erythrocytes. Results are given as the mean ± SD of three independent experiments.

As shown in Figure 6, DiMCh-AMP1 exhibited very low hemolytic activities against human erythrocytes. Even at 105.56 μM, DiMCh-AMP1 caused <5% hemolysis.

## Discussion

Although AMPs are regarded as promising candidates for pharmaceutical use, there are some limitations for their commercial development and clinical applications including potential toxicity, susceptibility to proteases, and high cost of peptide production. To overcome these challenges, various strategies have been developed of which peptide dimerization has received major consideration. Recent studies have shown that dimerization of AMPs increases their antimicrobial activity and selectivity (Güell et al., 2012; Hernandez-Gordillo et al., 2014; Lakshminarayanan et al., 2014). In our previous study, MCh-AMP1 was isolated from *M. chamomilla* and proven to be effective against of *Aspergillus* and *Candida* strains (Seyedjavadi et al., 2019). In this study, a dimeric peptide of MCh-AMP1 named DiMCh-AMP1 was designed and evaluated for antifungal activity and cytotoxicity. In this context, the stable and flexible linker that joins the independent segments without interfering with functionality is essential for the chimeric peptide regions of the new dimer to maintain its parental functions. In general, rationally designed hybrid AMPs with improved biological activities through the incorporation of flexible peptide linkers would contribute to a breakthrough in the technical limitations to the development of AMPs as substitutes for antibiotics. In the present study, several linkers were examined using computer programs to find the best linker to sustain functionality and retrieve the normal structure of the two parts of the recombinant protein (data are not shown in details). Finally, the flexible linker, GPDGSGPDESGPDES, was selected to connect the amino acid sequences together (MCh-AMP1-MCh-AMP1) and maintain their spatial configuration and activity. In a previous study, this linker was successfully used in a hybrid AMP LHP7, and the results showed enhancement of antimicrobial activity (Xi et al., 2013).

A cost-effective, large-scale production approach is required to commercialize dimeric peptides for industrial and medical applications. For this purpose, a variety of AMP expression systems were introduced by various researchers (Wu et al., 2008; Luan et al., 2014; Iglesias-Figueroa et al., 2016). In large-scale production systems, AMPs can now be produced in larger amounts following advances in the technology of recombinant DNA (Parachin et al., 2012). Protein expression in *E. coli* is relatively simple and less expensive compared to eukaryotic systems (Wang A. et al., 2010; Vriens et al., 2014). Eukaryotic cell expression requires a long production cycle, which leads to higher cost than microbial expression system (Farid, 2007; Chartrain and Chu, 2008). The *E. coli* expression system was applied to express recombinant DiMCh-AMP1 because of its well-studied genetic information, rapidly growing expression host, several cloning vector choices, easy culture, cost-effectiveness, and high product yield (Demain and Vaishnav, 2009). Moreover, as AMPs are toxic to host cells and sensitive to the proteolytic degradation of host intracellular proteins, the expression of such peptides in *E. coli* is not easy during synthesis; fusion protein has been shown to be a successful strategy to prevent toxicity and proteolysis of AMPs and to increase their expression levels in *E. coli* (Li, 2011). The pET system is a powerful system for the expression, purification, and detection of fusion proteins *in E. coli*. The mRNA structure has optimized based on low Δ*G* (d*G*) and energy of the start codon. This character could help ribosome binding and translation initiation. The 5′ terminus of the gene encoding the recombinant fusion protein DiMCh-AMP1 has folded in the way typical of all bacterial gene structures. The minimum free energy for secondary structures formed by RNA molecules was also predicted. Δ*G* of the best predicted structure was −66 kcal/mol. The obtained data demonstrated that the mRNA had the sufficient stability for the effective translation in the novel host.

Nevertheless, there are still inconveniences with inefficiency of the fusion protein cleavage or the high cost of the applied methods. Moreover, it is expensive or difficult to purify the protein/AMP expressed. The current study used a 22-residue N-terminal pro-peptide expression vector of pET28a(+), as a fusion encoding His-tag to produce AMPs and purify the protein. This plasmid was selected according to the manufacturer's recommendations and also the expression of a short-peptide recombinant as the scientific reference (Qiagen, 2003; Kumar and Sarma, 2010).

In the present study, CNBr was used to cleave condensed peptides, which had a very high efficacy and low cost, and the HPLC was then applied to purify cleaved DiMCh-AMP1. Previous studies reported that the AMPs released from fusion partners following CNBr digestion can cleave peptides at methionine residues (Hwang et al., 2001; Cheng et al., 2018). The yield of AMPs produced by recombinant technology completely depends on the peptide structure, vector type, digestion method, and the type of fusion proteins (Yang et al., 2012; Cheng et al., 2018). In the present study, recombinant technology was successfully used to produce DiMCh-AMP1 as a dimeric antifungal peptide with a yield ~0.9 mg/L of host cell culture in LB medium. Clement et al. (2015) reported recombinant production of Ba1 peptide in *E. coli* at a level of 900 μg/L in the exact similar conditions of vector and CNBr digestion to those of the present study.

Our results showed that the recombinant DiMCh-AMP1 antifungal peptide had stronger antifungal activities (MICs of 1.67–6.66 μM) than those of monomeric counterpart MCh-AMP1 (3.33–32 μM) reported earlier (Seyedjavadi et al., 2019). This finding is in accordance with a previous study that indicated the dimeric peptide KR-12 had higher antimicrobial activity than that of its monomeric counterpart (Gunasekera et al., 2020). These authors showed that KR-12 was effective against *C. albicans* as well as *Pseudomonas aeruginosa* and *Staphylococcus aureus*. The MIC of KR-12 for *C. albicans* was reported as 1.25 μM, whereas we reported MIC value of 3.33 μM against *C. albicans* for DiMCh-AMP1. They suggested that dimerization together with backbone cyclization is an effective strategy for improving both potency and stability of linear AMPs.

Our results showed that DiMCh-AMP1 with 36% total hydrophobic ratio had 4.3% hemolytic activity at the concentration of 105.56 μM, whereas parent MCh-AMP1 with the total hydrophobic ratio of 47% showed 10.6% hemolytic activity at the same concentration reported earlier (Seyedjavadi et al., 2019). The obtained results may be attributed to the fact that the penetration of highly hydrophobic peptides into the hydrophobic cores of red blood cell membranes is significantly higher, which leads to a stronger hemolysis during pore or channel formation (Chen et al., 2007).

The main antifungal mechanisms of action of the majority of antifungal peptides are increasing permeability, disrupting of microbial membranes, increasing the content of ROS in the cell, and generating the oxidative damage (De Cesare et al., 2020). In our previous study, MCh-AMP1 caused permeabilization of fungal cell membrane and induced ROS production in *C. albicans* (Seyedjavadi et al., 2020). With respect to the fact that our linker caused minimal structural changes in conformation of native peptide shown by the data from secondary and tertiary structure analyses, DiMCh-AMP1 may have a mechanism of antifungal action similar to the parent peptide. Nonetheless, to better characterize the DiMCh-AMP1, further works need to be carried out on the mode of action, stability, and effect on biofilm formation and other virulence factors in *C. albicans* and *Aspergillus* species.

## Conclusion

In the present study, an expression system based on *E. coli* as a host was successfully designed to produce the dimeric peptide DiMCh-MP1 with the aim of enhancing antifungal activity and reducing undesirable effects of the native peptide. Our results demonstrated that dimerization of MCh-MP1 to DiMCh-MP1 improved considerably the biological activity of the peptide by reducing its hemolytic activity and increasing its antifungal activity against both *Candida* and *Aspergillus* species. Taken together, these results clearly indicate that dimerized DiMCh-AMP1 could be considered as a potential candidate to proceed as a novel antifungal agent with enhanced antifungal activity and reduced cytotoxicity.

## Data Availability Statement

The original contributions presented in the study are included in the article/supplementary material, further inquiries can be directed to the corresponding author.

## Author Contributions

SS, JA, and MR-A conceived and designed the study, analyzed and interpreted the data, and were drafted and written the manuscript. SS, SK, AE, HH, RH, MG, MS-G, AI, JA, and MR-A performed the experiments. All authors approved the final version of manuscript. MR-A supervised the study.

## Conflict of Interest

The authors declare that the research was conducted in the absence of any commercial or financial relationships that could be construed as a potential conflict of interest.
